# Efficacy of naloxone in reducing postictal central respiratory dysfunction in patients with epilepsy: study protocol for a double-blind, randomized, placebo-controlled trial

**DOI:** 10.1186/s13063-016-1653-1

**Published:** 2016-11-03

**Authors:** Sylvain Rheims, Luc Valton, Véronique Michel, Louis Maillard, Vincent Navarro, Philippe Convers, Fabrice Bartolomei, Arnaud Biraben, Arielle Crespel, Philippe Derambure, Bertrand de Toffol, Edouard Hirsch, Philippe Kahane, Martine Lemesle Martin, Didier Tourniaire, Sébastien Boulogne, Catherine Mercier, Pascal Roy, Philippe Ryvlin, on behalf of ENALEPSY study group

**Affiliations:** 1 Department of Functional Neurology and Epileptology, Hospices Civils de Lyon, Lyon, France; 2 Lyon Neuroscience Research Center, INSERM U1028, CNRS UMR 5292, Lyon, France; 3 Epilepsy Institute (IDEE), Lyon, France; 4 Department of Neurology, University Hospital of Toulouse, Toulouse, France; 5 Department of Clinical Neurophysiology, University Hospital of Bordeaux, Bordeaux, France; 6 Department of Neurology, University Hospital of Nancy, Nancy, France; 7 Epileptology Unit, Assistance Publique-Hôpitaux de Paris - Groupe Hospitalier Pitié-Salpêtrière, Paris, France; 8 Brain and Spine Institute (ICM; INSERM UMRS1127, CNRS UMR7225), Pierre and Marie Curie University, Paris, France; 9 Department of Clinical Neurophysiology, University Hospital, Saint-Etienne, France; 10 Department of Clinical Neurophysiology and Epileptology, Timone Hospital, Marseille, France; 11 Department of Neurology, University Hospital of Rennes, Rennes, France; 12 Epilepsy Unit, University Hospital of Montpellier, Montpellier, France; 13 Department of Clinical Neurophysiology, Lille University Medical Center, EA 1046, Lille 2 University of Health and Law, Lille, France; 14 Department of Clinical Neurophysiology, INSERM U930, University Hospital of Tours, Tours, France; 15 Department of Neurology, University Hospital of Strasbourg, Strasbourg, France; 16 Department of Neurology, Michallon Hospital, Grenoble, France; 17 Institute of Neurosciences, INSERM U836, Grenoble Alpes University, Grenoble, France; 18 Department of Clinical Neurophysiology, University Hospital of Dijon, Dijon, France; 19 La Teppe Epilepsy Center, Tain l’Hermitage, France; 20 Department of Biostatistics, Hospices Civils de Lyon, Lyon, France; 21 Department of Clinical Neurosciences, Centre Hospitalier Universitaire Vaudois, Lausanne, Switzerland

**Keywords:** Epilepsy, SUDEP, Opioids, Naloxone

## Abstract

**Background:**

Generalized tonic-clonic seizures (GTCSs) are the main risk factor for sudden unexpected death in epilepsy (SUDEP). Experimental and clinical data strongly suggest that the majority of SUDEP results from a postictal respiratory dysfunction progressing to terminal apnea. Postictal apnea could partly derive from a seizure-induced massive release of endogenous opioids. The main objective of this study is to evaluate the efficacy of an opioid antagonist, naloxone, administered in the immediate aftermath of a GTCS, in reducing the severity of the postictal central respiratory dysfunction.

**Methods/design:**

The Efficacy of Naloxone in Reducing Postictal Central Respiratory Dysfunction in Patients with Epilepsy (ENALEPSY) study is a multicenter, double-blind, randomized, placebo-controlled trial conducted in patients with drug-resistant focal epilepsy who are undergoing long-term video-electroencephalogram (EEG) monitoring (LTM) in an epilepsy monitoring unit (EMU). We plan to randomize 166 patients (1:1) to receive intravenous naloxone (0.4 mg) or placebo in the immediate aftermath of a GTCS. Because inclusion in the study needs to take place prior to the occurrence of the GTCS, and because such occurrence is observed in about one-fourth of patients undergoing LTM, we plan to include a maximum of 700 patients upon admission in the EMU. The primary endpoint will be the proportion of patients whose oxygen saturation is <90 % between 1 and 3 min after the end of a GTCS. Secondary outcomes will include the following: the proportion of patients who show postictal apnea, the occurrence and duration of postictal generalized EEG suppression, the total duration of the postictal coma, postictal pain, and the number of patients who have a second GTCS within 120 min after the intravenous injection.

**Discussion:**

The demonstration of naloxone’s efficacy on the severity of postictal hypoxemia will have two primary consequences. First, naloxone would be the first and only therapeutic approach that could be delivered immediately to reverse postictal apnea. Second, demonstration that an opioid antagonist can effectively reduce postictal apnea would pave the way for an assessment of a preventive therapy for SUDEP targeting the same pathophysiological pathway using oral administration of naltrexone.

**Trial registration:**

ClinicalTrials.gov identifier: NCT02332447. Registered on 5 January 2015.

**Electronic supplementary material:**

The online version of this article (doi:10.1186/s13063-016-1653-1) contains supplementary material, which is available to authorized users.

## Background

Sudden unexpected death in epilepsy (SUDEP) is defined as sudden, unexpected, witnessed or unwitnessed, nontraumatic, and nondrowning death in patients with epilepsy, with or without evidence of a seizure and excluding documented status epilepticus, in which postmortem examination does not reveal a toxicologic or anatomic cause of death [1]. SUDEP primarily affects young adults with drug-resistant epilepsy, with an incidence of about 0.4 %/year [2]. Among the SUDEP risk factors that have been individualized so far [2], the presence and frequency of generalized tonic-clonic seizures (GTCSs), either primary or secondary generalized), were found to represent the main ones, with an OR >15 for patients with three or more GTCSs per month [3].

There is currently no effective treatment to prevent SUDEP, apart from optimizing antiepileptic drugs (AEDs) [2, 4]. As underscored by the World Health Organization, there is an urgent need to develop specific therapeutic approaches to tackle this issue [5]. Considering that patients with the highest risk of SUDEP are young adults who have drug-resistant epilepsy with frequent GTCSs, it has been suggested that research on SUDEP prevention should be focused on this specific population [3, 6].

Although the exact pathophysiologic mechanisms that lead to SUDEP remain unknown [2, 7], most of the evidence lends support to the predominant role of central respiratory dysfunction [8]. Experimental and clinical data strongly suggest that most SUDEP results from a postictal respiratory dysfunction progressing to terminal apnea, followed by cardiac arrest [8, 9]. In addition, postictal generalized electroencephalogram (EEG) suppression (PGES), which is another factor associated with SUDEP [10], might primarily represent an ancillary marker of profound postictal hypoxemia [11]. Importantly, the central respiratory dysfunction that leads to SUDEP typically occurs between 1 and 3 min after the end of a GTCS [9], supporting the view that central respiratory dysfunction might be at least partly triggered by the release of neurotransmitter involved in seizure termination [8]. In this context, there might be a short time window during which this mechanism might be reversed by an appropriate treatment [8].

Animal studies suggest that seizure-related release of endogenous opioid peptides participate in termination of seizures [12]. In patients with epilepsy, functional imaging studies have confirmed that seizures induce release of endogenous opioids [13, 14]. The brainstem respiratory centers contain the highest density in opioid receptors [15], accounting for respiratory depression being one of the cardinal symptoms of opioid overdose [16, 17].

Our hypothesis is that SUDEP results partly from postictal apnea promoted by a GTCS-induced massive release of endogenous opioids, and that an opioid antagonist could represent an effective preventive treatment for SUDEP [8]. This could be achieved by chronic administration of naltrexone, an opioid antagonist that has been used in a large population of patients with chronic alcoholism at high risk of seizures, without showing any proconvulsive effect. This is a crucial feasibility issue because antagonizing a mechanism thought to participate in seizure termination could theoretically aggravate seizures.

Before evaluating the efficacy of chronic administration of naltrexone, it is legitimate to perform a proof-of-concept study by testing the acute effect of an equivalent injection treatment (naloxone) in the immediate aftermath of GTCS recorded in the hospital during video-EEG monitoring of patients with refractory epilepsy. One-third of these patients develop postictal respiratory dysfunction and hypoxemia [18], which should be reduced by our intervention if our hypothesis is correct.

## Methods/design

**Fig. 1:**
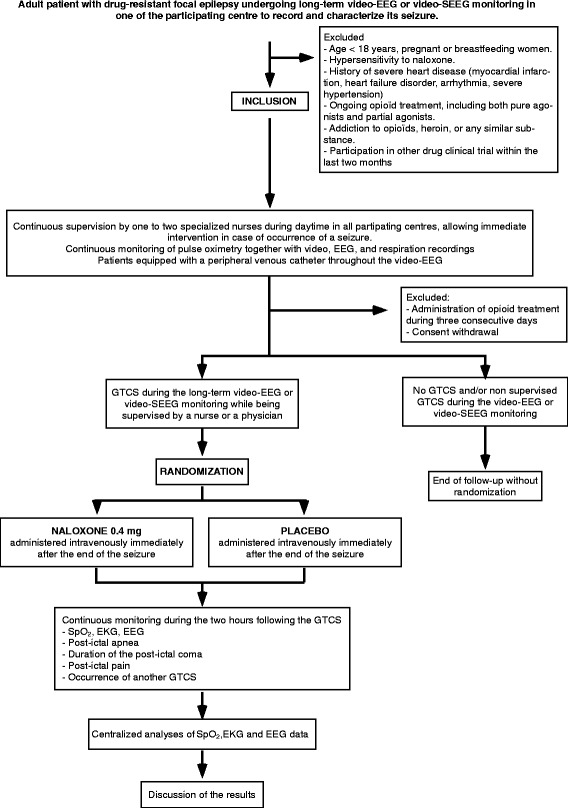
Flowchart of the trial. *EEG* Electroencephalogram, *EKG* Electrocardiogram, *GTCS* Generalized tonic-clonic seizure, *SEEG* Stereoelectroencephalography, *SpO*_*2*_ Peripheral oxygen saturation measured by pulse oximetry

The Efficacy of Naloxone in Reducing Postictal Central Respiratory Dysfunction in Patients with Epilepsy (ENALEPSY) study is a multicenter, double-blind, randomized, placebo-controlled trial (Fig. 1 and Additional file 1: Standard Protocol Items: Recommendations for Interventional Trials [SPIRIT] checklist). The main objective of the study is to evaluate the efficacy of 0.4 mg intravenous naloxone versus placebo administered in the immediate aftermath of a GTCS in reducing the severity of the postictal central respiratory dysfunction occurring 1–3 min after the end of the seizure, as measured by peripheral oxygen saturation measured by pulse oximetry (SpO_2_).

### Study setting

The study will be conducted with patients who have drug-resistant focal epilepsy investigated with long-term scalp video-EEG or video-stereoelectroencephalography (video-SEEG) in 1 of the 15 participating epilepsy monitoring units (EMUs) in France (see Additional file 2). The modalities of the video-EEG monitoring and patient management will be consistent with current practices and will be similar across all participating centers, including modalities of invasive recording using SEEG in the ten EMUs that perform both video-EEG and video-SEEG monitoring. Specifically, all of them undergo systematic recording of SpO_2_ coupled with video-EEG. The reliability of the measurement of SpO_2_ during seizures has been validated by two studies in which researchers carried out this type of monitoring [18, 19].

### Study duration

Recruitment into the trial commenced in July 2015 and is anticipated to be completed in July 2018. The duration of participation of each patient in the trial will be a maximum of 36 days. Long-term video-EEG/video-SEEG monitoring typically lasts between 7 and 15 days, and occasionally up to 21 days. However, the total duration of monitoring will be variable across patients, defined by clinical parameters, specifically the occurrence of a sufficient number of seizures to allow conclusions to be drawn about the localization of the seizure onset zone. For safety reasons, a phone visit will be held 15 days after the end of the monitoring.

### Participants

About 25 % of patients with drug-resistant partial epilepsy who are undergoing long-term video-EEG monitoring develop at least one focal secondary generalized tonic-clonic seizure (sGTCS) [18, 20, 21]. However, these patients cannot be individualized a priori. Therefore, all adult patients with drug-resistant epilepsy who will undergo long-term video-EEG monitoring in one of the participating centers will be included in the study according to the following inclusion criteria:

- Adult patients (≥18 years) with drug-resistant focal epilepsy
- Patients undergoing long-term video-EEG or video-SEEG monitoring in one of the participating centers to record and characterize their seizures
- Patients who provide written informed consent to participate in the study
- Patients affiliated with the French health care system

Patients fulfilling one or more of the following exclusion criteria will not be eligible for inclusion:

- Children (<18 years)
- Pregnant or breastfeeding women
- Hypersensitivity to naloxone
- History of severe heart disease (i.e., myocardial infarction, heart failure disorder, arrhythmia severe hypertension)
- Ongoing opioid treatment, including both pure agonists and partial agonists
- Addiction to opioids, heroin, or any similar substance
- Participation in another drug clinical trial within the last 2 months

Patients who have an sGTCS during the long-term video-EEG or video-SEEG monitoring while being supervised by a nurse or a physician will be randomized to receive intravenous naloxone (0.4 mg) or placebo.

As indicated in the exclusion criteria, naloxone will not be allowed for patients receiving ongoing opioid treatment, including both pure agonists and partial agonists. Therefore, patients receiving ongoing opioid treatment will be excluded. However, in the context of presurgical evaluation, especially for patients who undergo invasive recording with SEEG, the management of pain might require temporary administration of an opioid agonist. In such patients, short-lasting administration of opioid treatment following inclusion will be authorized, except for extended-release formulations of morphine. However, this will result in temporary ineligibility for randomization during the 12 h following the last opioid administration. If pain management requires administration of opioid treatment during 3 consecutive days, the patient will be excluded from the study.

### Interventions

Naloxone is a specific opioid antagonist without partial agonist activity. Intravenous administration has been approved in France since 1977 for the treatment of acute opioid overdose. In this indication, the recommended starting dose is 0.4 mg [16]. In acute opiate overdose, the delay between intravenous injection and awakening ranges from 30 s to 2 min, and its apparent duration of action is 20–90 min [16]. No epilepsy-related alert has been reported for this product. Naloxone administration will consist of naloxone hydrochloride dihydrate (0.4 mg/ml) packaged in 1-ml vials that can be intravenously administered to the patient without additional dilution. The placebo will be isotonic sodium chloride with preparation in 1-ml vials.

### Blinding and randomization

To avoid any follow-up or measurement bias, the patient, the supervising nurse, or the physician and the investigator will not be aware of the nature of the administered treatment. To respect the blinding and to ensure indistinguishability, preparations of both naloxone and placebo will be centralized by the pharmaceutical department of Hospices Civils de Lyon. The study drugs will then be distributed to the pharmacies of other investigator sites and stored at room temperature. Because of urgent need of treatment in case of occurrence of GTCS, one or more study treatments will be stored at room temperature in each EMU according to the expected number of randomizations at each center

Randomization will be centralized, stratified by center, and balanced by block of patients. For each study center, randomization lists will be prepared by the clinical research unit of Hospices Civils de Lyon. The pharmaceutical department of Hospices Civils de Lyon will prepare numbered batches according to the randomization lists.

In case of the occurrence of a GTCS, the affected patient will be randomized in a 1:1 ratio and assigned to the naloxone group or the placebo group. The batch number will be registered in the patient’s case report form. All digital data (video, EEG, respiration, SpO_2_) will be centralized and evaluated in blinded fashion with other data by the coordinator investigator of the study, who will not be involved in the video-EEG monitoring of the included patient.

If case of occurrence of a serious adverse event, the Antipoison and Toxicovigilance Center of Lyon will proceed to unblinding 24 h/24 h upon request by the study coordinator, the investigators, or the sponsor. A detailed written procedure for unblinding will be provided to all persons involved.

### Study conduct

All included patients will benefit from continuous monitoring of SpO_2_ and will be have a peripheral venous catheter inserted throughout the video-EEG/video-SEEG. During video-EEG/video-SEEG, patients are continuously supervised by one or two specialized nurses during the daytime at all participating centers. This continuous supervision allows immediate intervention in case of occurrence of a seizure. In six centers, patients are supervised by one specialized nurse during the night, whereas they are not supervised in the other centers. Given this organization of some EMUs, a GTCS might occur during a period without supervision. Unsupervised GTCS will not result in randomization, and the patient will remain eligible for randomization in case of occurrence of another GTCS during the monitoring. Inclusion in the study will not modify antiepileptic drug withdrawal, the management of which will remain at the discretion of each center.

In case of occurrence of a GTCS, patients will be randomized (1:1) into one of two groups: a single dose of 1 ml of either (1) naloxone or (2) placebo will be administered by the supervising nurse or physician, using direct intravenous injection. The evolution from a focal seizure to a GTCS being gradual, and the total duration of the seizure ranging from 2 to 3 min, the injection will be prepared during the course of the seizure. Given the assumptions about the role of endogenous opioid release in the spontaneous termination of seizures, naloxone will be administrated immediately after the end of the GTCS and not before. Specifically, treatment administration will be performed within the 2 min following the end of the GTCS.

Immediately after the administration of the treatment, the supervising nurse or physician will monitor respiratory movements to detect absence of chest expansion, SpO_2_, heartbeat and blood pressure, recovery of consciousness and pain, using a visual analogue scale. In patients with SpO_2_ < 85 % during >2 min after the injection of the study drug, O_2_ will be administered using a high-concentration breathing mask (15 L/min). Occurrence of one or more GTCSs during the 2 h following treatment administration will be monitored.

All digital data (video, EEG, respiration, SpO_2_) will be centralized and evaluated blind to other data. An automatic and objective analysis of SpO_2_ data will be performed using a specific detection algorithm that allows automatic detection and quantification of SpO_2_ and electrocardiogram variations.

### Outcome measures

#### Primary endpoint

The primary endpoint is the proportion of patients whose SpO_2_ is <90 % during at least 5 s between 30 s and 5 min after the onset of intravenous injection of the study drug in the immediate aftermath of a GTCS. The delay between the end of the seizure and administration of the treatment will be precisely determined by the video recording of the event, given that the nurse or physician who will administer the study drug will be required to both loudly order “injection” and raise an arm at the onset of injection. Previous studies have shown that the measurement of SpO_2_ is reliable in the vast majority of seizures, including GTCSs [18, 19]. To eliminate artefactual changes of SpO_2_, however, the criterion of hypoxemia duration of at least 5 s is required.

#### Secondary endpoints

##### Efficacy endpoints

- Number of patients who show postictal apnea, defined as the absence of chest expansion during a period >10 s between 1 and 3 min after the end of the GTCS
- Number of patients for whom O_2_ administration is required within the 10 min following the end of a GTCS
- Number of patients for whom cardiorespiratory rescue procedure is required within the 10 min following the end of a GTCS
- Total duration of the postictal generalized EEG suppression, defined as lack of detectable EEG activity >10 μV in amplitude on all leads [10]
- Total duration of the postictal coma, defined as the delay between the end of the seizure and the recovery of consciousness assessed by the ability to meet one single verbal command (handshake)

##### Safety endpoints

- Report of adverse events observed throughout the study
- Assessment of pain using a visual analogue scale immediately after recovery of consciousness following the postictal coma.
- Number of patients who have a second GTCS within 120 min after the intravenous injection

### Data monitoring

A clinical research assistant mandated by the sponsor will ensure the successful completion of the study and the collection of data generated by writing, documentation, recording, and report, in accordance with the standard operating procedures implemented at the Hospices Civils de Lyon and in accordance with good clinical practice and regulatory legislation. An independent Data and Safety Monitoring Board (DSMB) external to the trial investigators has been established specifically to monitor data throughout the study to determine if it is appropriate, from both scientific and ethical standpoints, to continue the study as planned. The DSMB will meet every year to review serious adverse events and propose continuing or stopping the study. The DSMB is made up of three experts in epilepsy (A. Rossetti, Department of Clinical Neurosciences, Centre Hospitalier Universitaire Vaudois, Lausanne, Switzerland), anesthesiology (Dr. A. Charton, Strasbourg University Hospital, Strasbourg, France), and methodology in clinical research (S. Chabaud, Centre Leon Berard, Lyon, France).

### Sample size

Sample size was determined using Fisher’s exact test with a two-sided alternative hypothesis. For a significance level of 5 % (two-tailed), assuming a proportion of patients whose SpO_2_ is <90 % during at least 5 s between 30 s and 5 min after the onset of intravenous injection of the study drug in the immediate aftermath of a GTCS of 33 % in the control group [18] and 11 % in the naloxone group, 69 patients should be included in each group to reject the null hypothesis in 90 % of cases, with a 95 % CI ranging from 0.077 to 0.547. With a proportion of unusable records for technical reasons of 20 % being expected, 166 patients will be randomized. Because we anticipate that 25 % of patients eligible for inclusion will have a sGTCS during long-term video-EEG monitoring, we will need to include 700 patients.

### Statistical analyses

The primary analysis will be based on the intention-to-treat principle, including all randomized patients with usable records for SpO_2_. The proportion of patients whose SpO_2_ is <90 % during at least 5 s between 30 s and 5 min after the onset of intravenous injection of the study drug in the immediate aftermath of a GTCS will be presented in each group (naloxone and placebo) with the corresponding exact 95 % CI. These proportions will be compared between the two groups using Pearson’s chi-square test (or Fisher’s exact test if conditions for the chi-square test are not fulfilled).

A per-protocol analysis of the primary endpoint will also be performed in the population, which will comprise all randomized patients with usable records for SpO_2_ having no major protocol deviation such as patients in whom the onset of intravenous injection of the study drug was within the 2 min following the end of the seizure (whereas those in whom the study drug was administered >120 s after the end of the seizure will be excluded). Secondary endpoints will be compared between groups using Pearson’s chi-square test, Fisher’s exact test, Student’s *t* test, or the Wilcoxon-Mann–Whitney test, depending on the variable studied. The significance level will be set at 0.05.

## Discussion

Although SUDEP is the leading cause of death in people with chronic refractory epilepsy, there is currently no effective treatment to prevent SUDEP, apart from optimizing AEDs [2, 4]. As underscored by the World Health Organization [5], there is an urgent need to develop specific therapeutic approaches to tackle this issue. Given the pathophysiologic link between the occurrence of central apnea in the aftermath of GTCS and the risk of SUDEP, treatment strategies aimed at reducing the severity of postictal respiratory dysfunction appear to be the most promising way to prevent SUDEP [6, 8].

The demonstration of naloxone’s efficacy in reducing the severity of postictal hypoxemia will have two primary consequences. First, naloxone would be a therapeutic approach that could be immediately delivered to reverse postictal apnea, especially during long-term video-EEG monitoring. Although rare, SUDEP has occurred during video-EEG in several EMUs in Europe [9]. In addition, the availability of employing the intramuscular route for naloxone [16] renders possible its use at home, especially for patients in whom severe postictal hypoxemia would have been observed in the hospital. By reducing postictal apnea, naloxone might also shorten the postictal phase and promote quicker recovery of consciousness.

Second, a demonstration that an opioid antagonist can effectively reduce postictal apnea would pave the way for an assessment of a preventive therapy targeting the same pathophysiologic pathway using naltrexone. Indeed, this orally administered opioid antagonist can be delivered over the long term with an excellent safety profile, including in patients undergoing alcohol withdrawal at high risk of seizure, in whom a proconvulsive effect has not been observed [22, 23]. Following this naloxone study, the impact of chronic naltrexone therapy might first be tested for postictal hypoxemia during video-EEG (by treating patients chronically before and during their video-EEG monitoring). If this second study proved positive, a large-scale study with ambulatory patients could then be undertaken to test the impact of chronic naltrexone treatment on the risk of SUDEP in high-risk populations [8].

### Trial status

The study protocol was designed in September 2013 and funded by the French Ministry of Health in 2014. The study was registered with ClinicalTrials.gov (NCT02332447) in January 2015. The first participant was enrolled on 26 June 2015. A total of 157 patients have been included so far, of whom 16 have been randomized.

## Additional files

Additional file 1: Standard Protocol Items: Recommendations for Interventional Trials (SPIRIT) 2013 checklist: recommended items to address in a clinical trial protocol and related documents. (DOC 125 kb)
Additional file 2: List of investigators in the ENALEPSY study. (DOC 201 kb)

## Abbreviations

AED: Antiepileptic drug
DSMB: Data and Safety Monitoring Board
EEG: Electroencephalogram
EKG: Electrocardiogram
EMU: Epilepsy monitoring unit
ENALEPSY: Efficacy of Naloxone in Reducing Postictal Central Respiratory Dysfunction in Patients with Epilepsy trial
GTCS: Generalized tonic-clonic seizure
LTM: Long-term video-electroencephalogram monitoring
PGES: Postictal generalized electroencephalogram suppression
SEEG: Stereoelectroencephalography
sGTCS: Secondary generalized tonic-clonic seizure
SPIRIT: Standard Protocol Items: Recommendations for Interventional Trials
SpO_2_: Peripheral oxygen saturation measured by pulse oximetry
SUDEP: Sudden unexpected death in epilepsy
