# Supplementary material for: Efficacy of naloxone in reducing postictal central respiratory dysfunction in patients with epilepsy: study protocol for a double-blind, randomized, placebo-controlled trial
Source: Trials. 2016 Nov 3;17:529. doi: 10.1186/s13063-016-1653-1 (PMC5094038; doi:10.1186/s13063-016-1653-1)
Supplement: Additional file 2: — List of investigators in the ENALEPSY study. (DOC 201 kb) [file 13063_2016_1653_MOESM2_ESM.doc]

**List of investigators of the ENALEPSY study**

Bordeaux: Drs V. Michel, M. De Montaudouin, C. Marchal

Clermont-Ferrand: Dr D.S Rosenberg

Dijon: Dr M. Lemesle

Grenoble: Pr P. Kahane, Drs L. Minotti, L. Vercueil, AS Jobst

La Teppe: Dr J. Petit, Dr D. Tourniaire, Dr V. Eid, Dr P. Latour

Lille: Pr P. Derambure, Dr W. Szurhaj,

Lyon: Dr S. Rheims, Dr H. Catenoix, Dr N. Andre-Obadia, Dr J. Isnard, Dr A. Montavont, Dr S. Boulogne

Marseille: Pr F. Bartolomei, Drs. A.Trébuchon, A. Mc Gonigal, S. Aubert

Montpellier: Drs A. Crespel, P. Gelisse, B. Mercedes

Nancy: Pr L. Maillard, Pr L. Tyvaert Dr JP Vignal

Paris Pitié-Salpêtrière: Prs V. Navarro, S. Dupont, Dr C. Adam, Dr V-H Nguyen-Michel, Dr M Damiano, Dr V Lambrecq

Rennes: Drs A. Biraben, A. Nica

Saint-Etienne: Dr P. Convers, Dr L. Mazzola

Strasbourg: Pr E. Hirsch, Dr MP. Valenti, Dr J. Scholly, Dr C. Behr

Toulouse: Drs L. Valton, M. Denuelle, J. Curot

Tours: Pr B. De Toffol
